# Advances in Penile Prosthesis Surgery: A Clinical Update

**DOI:** 10.5152/tud.2020.20442

**Published:** 2020-10-23

**Authors:** Ömer Onur Çakır, Wai Gin Lee, Philippa Ralph, Mathew Megson, David J. Ralph

**Affiliations:** 1 Institute of Andrology, University College London Hospital, London, UK; 2 Division of Surgery and Interventional Science, University College, London, UK

**Keywords:** Erectile dysfunction, penile implant, penile prosthesis, surgery

## Abstract

Despite the introduction of effective oral pharmacotherapy for the treatment of erectile dysfunction, penile implants are still the standard care for patients who do not respond well to medical therapy. Since the first inflatable penile implant surgery was performed almost 40 years ago, a variety of improvements in the penile prosthesis design, and advancements in material science, surgical technique, and post-operative care have been developed to increase patients’ satisfaction, as well as that of their partners. Penile implants have evolved vastly during that same time frame and now represent the cutting-edge technology, durability, and function. Here, advancements are reviewed with a focus upon recent developments in surgical techniques and device technology.

**Main Points**

- Penile implants have remained the gold standard for therapy for men with erectile dysfunction refractory to pharmacotherapy for decades.
- Here, advancements are reviewed with a focus upon recent developments in the penile prosthesis design, material science, device technology, and surgical techniques.
- Future research to develop a perfect penile prosthesis will mainly focus on easy-to-activate and user-friendly devices, simplifying implantation surgery techniques, simulating the physiological erection, minimizing post-operative infection risks, and enhancing the durability of device materials.

## Introduction

Penile implants have remained the gold standard for therapy for men with erectile dysfunction (ED) refractory to pharmacotherapy for decades.^1^ The primary aim of penile implant insertion is restoration of normal erectile function to allow successful penetrative sexual intercourse. The American Urological Association recommends that all men with ED be informed about penile implant options as a potential and effective treatment modality.^2^ In addition, both patient and partner satisfaction rates are reported to be the highest with penile prosthesis compared with oral medical therapies.^3^ Although highly effective pharmacotherapy options and potential future medical treatments (e.g., low-intensity shock wave therapy and stem cell therapy) are available in the market for ED treatment, penile prostheses will likely remain an important option with the highest satisfaction rate.

As penile prosthesis technology improved and became more widely used, its indications expanded to treat concomitant disorders, such as Peyronie’s disease (PD) and priapism with concomitant ED.^4^ In patients with PD, the inflatable penile prosthesis (IPP) is inflated and the corpora are then modeled around the device to correct the deformity. Extended cases of ischemic priapism may also be treated with a penile implant. For example, ischemic priapism lasting longer than 72 hours invariably results in severe ED, but early placement of a penile prosthesis within a few days may treat the irreversible ED and priapism before severe fibrosis develops.^5^

Since their introduction as a patient treatment 40 years ago, penile prostheses have undergone myriad improvements and advancements in material science, technology, and design. The invention of the IPP heralded a new era for device innovation,^6^ which led to a cascade of new devices in subsequent years. Two- and threepiece IPPs allowed for individualized device selection that incorporated patient-specific anatomic considerations.^6^ Expansile materials for cylinder design have been refined to improve durability while maximizing penile length and girth.^7^

More durable implant components and antibiotic or hydrophilic coatings minimize the overall infection risk. Penile prostheses impregnated with antibiotics were shown to reduce patients’ post-operative infection risk significantly following implantation.^8^ In addition, advances in hydrophilic coating technology have increased the absorption level of aqueous antibiotic solutions, which has helped to decrease bacterial adherence on the prosthesis surface, which also reduced risks of post-operative infection due to interactions between pathogens, hosts, and biomaterials.^8,,9^

Other major innovations in device production technology significantly reduced mechanical failure rates. For example novel lock-out valve systems have virtually minimized the risk of auto-inflation, which occurs when the implant cylinders can no longer fully deflate and a partial or permanent erection occurs.^10^ Moreover advances in tubing have reduced leakage risks at connection points; for example, novel kink-resistant tubing materials and preassembled implants that require only minimal splicing for the tubing.^11^ Evolution of pump designs has also optimized the patient experience, such as the easier-to-operate one-touch release pump systems.^12^

Parallel to technological enhancements in the prosthesis systems and material design, a myriad of evolutionary surgical techniques have emerged to achieve better cosmetic results, improved patient safety, and reduced infection risks; these include no-touch approach, ectopic reservoir placement, and scrotoplasty.^13^ Novel supplementary instruments, such as Furlow devices and low-profile reservoirs, have helped facilitate the implant insertion and reservoir placement processes.^14^

The past few decades have seen a variety of new advancements in penile prosthesis surgery, in terms of innovative devices, material designs, and the development of novel surgical techniques. In this review, we highlight the aforementioned advancements in penile implant surgery and discuss novel directions in penile prosthesis technology.

## Clinical Update

### Advances in Penile Prosthesis Technology and Design

Conventional IPPs carry potential risks of infection, mechanical malfunction, and erosion. Indeed, improvements in pros thesis technology have significantly reduced the risk of device malfunction within 5 years to less than 2%.^11^ In addition, when considering the patient’s experience, these traditional IPPs do not mimic an endogenous physiological erection. They still require patient or partner manipulation, and although fairly visibly discrete, they have palpable components that affect the couple’s perception of discretion.^11^

The most critical complication of penile prosthesis implantation surgery is infection. Historically, the rate of infection for primary penile implants has been reported between 1%-3% for primary implants and up to 10% for implants undergoing revision or replacement.^15^ The formation of a biofilm on the prosthetic’s surface plays a crucial role in the development of postoperative infection.^16^ Bacterial contamination of the device may occur prior to, during, or after the operation.^11^ Establishment of a biofilm on the implant occurs when bacteria to establish micro-colonies through clonal expansion.^17^ Biofilms are a serious challenge because they render their microbial colonies impenetrable to antibiotic therapy. Antibiotic coatings are a well-known highly effective innovation that inhibit microbial colonies from becoming established.^11^ This coating can be pre-coated into the implant itself by the manufacturer or bound by immersion of the hydrophilic implant into an antibiotic solution by the surgeon prior to insertion. This intervention has led to the current 1%-3% rate of infection, which is lower in the context of high-volume surgeons.^15^

A fascinating innovation in the management of biofilm-related prosthesis infection is the application of ultrasound targeted microbubble destruction (UTMB) as an intervention.^11^ The efficacy of this revolutionary intervention has been shown for management of *Staphylococcus epidermidis* biofilms *in vitro* and in animal models.^18^ Administration of UTMB in conjunction with vancomycin therapy has demonstrated synergy between these 2 means of treatment.^19^ The peptide human β-defensin 3 has also been shown to have efficacy in the destruction of Staphylococcal biofilms, particularly when administered in conjunction with UTMB.^20^ Although a majority of studies on this topic have focused on orthopedic prostheses composed of titanium, UTMB may be of interest in the management of penile prosthesis infections and warrants further study.^20^

If an implant becomes infected, then international consensus guidelines recommend immediate removal of the implant, followed by irrigation of the surgical area with broad spectrum antibiotics.^7^ Unfortunately, if another implant is not placed immediately, a severe corporal fibrosis associated with dense scar tissue formation occurs, followed by penile shortening. Recently, an innovative alternative solution to prevent the aforementioned complication was reported by Swords and colleagues,^21^ which is a synthetic plaster-like vancomycin/tobramycin internal cast.

This calcium sulfate cast, impregnated with antimicrobials, is placed inside the infected corporal space as a temporary “placeholder” to facilitate clearance of potential bacteria. This cast also has an additional preventive effect on penile shortening via preserving the intracorporal space for a future penile prosthesis implantation.^21^ However, the role of this novel approach in implant infection remains unclear. Further research will be required for this to be considered as a standard care.

Important innovative improvements in the mechanics and material design of penile prosthetics have also been announced, parallel to novel technological developments. The mechanism of action for conventional IPP’s is based on simple hydraulic principles. Although this hydraulic mechanism is adequate, it relies on pressure and a reservoir that is connected to the cylinders by a tubing system. Unfortunately, this particular design is prone to leakage and other mechanical malfunctions. Valves are also needed to control the flow and direction of fluid, as well as to resist forces encountered in normal use, which is prone to autoinflation even if it contains a lock-out valve.^22^

However, material science undergoes constant improvement, with newer substances invented and introduced on a regular basis. In this context, new penile prosthetics rely on particular material designs, in terms of the expansile and contractile metal alloys to mimic the physiological rigid erection, which allows for successful penetrative sexual intercourse. Heat-sensitive polymers that change conformation and rigidity have also been explored for the construction of penile implants. For example, a particular prosthesis type designed by Le and colleagues^23^ has a heat-activated nickel-titanium-based shape memory alloy, which alternates between a flaccid to erect configuration solely by the application of a heating pad and from erect to flaccid phase by application of an ice pack. This particular prosthesis is able to produce the mechanical forces needed to reach a rigid erection sufficient for successful penetrative sexual intercourse, and it is comparable with that produced by traditional hydraulic-based penile implants.^23^ The same research group also recently introduced a touchless prosthesis designed to achieve a set shape from magnetic induction.^24^ This novel prosthesis was implanted in an animal model and in several cadavers in the “flaccid” state and then activated within 45 seconds using an external magnetic inducer wand. The researchers reported rigid erection necessary for penetration and the implant was also able to resist substantial buckling forces.^24^ Although early in development, the potential of such a device to eliminate the need for pumps and hydraulic tubing may pose a substantial advantage in terms of durability and ease of operation.

Another innovative penile implant device (Zephyr FTM) was produced in Switzerland for phalloplasty patients. It has both malleable and inflatable variants, which provide physiological erection sufficient for penetrative sexual intercourse in this particular patient population.^25^

### Advances in Operation Technique

Although penile implant technology and material designs have shown a significant advancement parallel to technological improvements, surgical implantation procedures continue to be complex and only highly specialized urological surgeons undertake these particular procedures with regular frequency. In this context, surgical outcomes appear to improve with high-volume implanters in high-volume centers.^22^

Preservation of penile length is important to both the patient and the partner. Penis length and size is cognitively related to the level of masculinity for a majority of men. Thus, many patients expect to have the same penile girth and length that they had before their prosthesis surgery. Men with decreased penile length and girth report higher rates of dissatisfaction and a decreased quality of life.^26^ As a result, surgical enhancement techniques combined with penile prosthesis surgery have been introduced to preserve penile length.^27^ In this context, release of the penoscrotal web (ventral phalloplasty) in combination with prosthetic implantation surgery is an advanced technique, which enhances the patient perception of increased penile length and is reported to increase the patient and partner satisfactions.^28^ In addition, dorsal phalloplasty has also been described recently to increase visible penis length. This approach is based on using permanent sutures to tack the dermis and pre-pubic fat to the symphysis pubis, which achieves a 23% increase in penile length.^29^

Another technique for preventing penile shortage due to penile implant insertion was introduced in the current decade, known as the sliding technique.^30^ Although the method was later revised into the modified sliding technique (MoST) and the multiple-slit technique, a majority of implant surgeons avoid this approach currently because of a high risk for glans necrosis due to aggressive neurovascular bundle and urethra mobilization.^31^ However, recently, a novel MoST was introduced by Egydio, which significantly reduces the glans necrosis complication in the previous sliding methods.^32^ In this new approach, tunica defects created during the sliding procedure are not covered with a graft and multiple smaller tunical cuts are preformed rather than 2 large cuts. This technical difference decreases the risk of post-operative cylinder bulging complications and it also protects the penile girth.^32^

An advanced novel modified glanulopexy technique was introduced recently for correcting post-operative supersonic transporter deformity and glandular hypermobility complications in men undergoing penile implant surgery implantation.^33^ This new technique is performed through a small incision that avoids unnecessary manipulation of Buck’s fascia, which reduces the risk of post-operative altered penile sensation.^33^

### What is Next?

We believe that future research to develop a perfect penile prosthesis will mainly focus on easy-to-activate and user-friendly devices, simplifying implantation surgery techniques, simulating the physiological erection, minimizing post-operative infection risks, and enhancing the durability of device materials. Future penile implant mechanics that rely on artificial intelligence systems controlled by electromagnetic signals, or even by the central nervous system, is no longer a dream in our technologically driven era. Because of ongoing improvements in material science, it will not be a surprise in the future to see durable and infection-resistant prosthetic devices that avoid mechanical failure, that do not require surgical revision, and that will mimic a physiological erection. In addition, maybe these novel devices will be implanted easily by simple surgical techniques even in an out-patient setting.

## Conclusion

Penile implants have been used for decades as crucial components of ED treatment and they are still the gold standard for patient’s refractory to medical therapy. A myriad of advances and innovations have been introduced parallel to current technological improvements, which have led to a better device durability, higher patient satisfaction, and lower post-operative complications. Future advances in material science and innovations in prosthesis design, along with novel improvements in surgical techniques, should be seen by future researchers as a good opportunity to achieve an ideal implant.
